# Sexual Activity before Sports Competition: A Systematic Review

**DOI:** 10.3389/fphys.2016.00246

**Published:** 2016-06-21

**Authors:** Laura Stefani, Giorgio Galanti, Johnny Padulo, Nicola L. Bragazzi, Nicola Maffulli

**Affiliations:** ^1^Sports Medicine Center, School of Sports Medicine, Department of Experimental and Clinical Medicine, University of FlorenceFlorence, Italy; ^2^University eCampusNovedrate, Italy; ^3^Faculty of Kinesiology, University of SplitSplit, Croatia; ^4^Department of Health Sciences, School of Public Health, University of GenoaGenoa, Italy; ^5^Department of Musculoskeletal Disorders, Faculty of Medicine and Surgery, University of SalernoSalerno, Italy; ^6^Centre for Sports and Exercise Medicine, Barts and The London School of Medicine and Dentistry, Queen Mary University of LondonLondon, UK

**Keywords:** athletes, abstinence, circadian rhythm, competition, performance, sex, time of day

## Abstract

Sexual activity before competition has been considered as a possible cause for reduced performance since ancient Greece and Rome. Recently, the hypothesis that optimal sport performance could be influenced by a variety of factors including sexual activity before competition has been investigated. However, few scientific data are available, with the exception of anecdotal reports of individual experiences. The present systematic review focused on the current scientific evidence on the effects of sexual activity on sport performance regardless of sport type. Data were obtained following the Preferred Reporting Items for Systematic Reviews and Meta-analyses (PRISMA) guidelines, using PubMed/MEDLINE, ISI/Web of Science, the Cochrane Collaboration Database, Cochrane Library, Evidence Database (PEDro), Evidence Based Medicine (EBM) Search review, National Guidelines, ProQuest, and Scopus, all searched from inception further, to broaden the search, no time filter nor language restriction have been applied. Also, the gray literature was mined using Google Scholar. Only relevant scientific articles reporting outcomes of athletic performance after sexual activity were considered. The impact of sexual activity before a sport competition is still unclear, but most studies generally seem to exclude a direct impact of sexual activity on athletic aerobic and strength performance. The most important aspect seems to be the interval from the time of the sports competition that affects negatively the performance if it is shorter than 2 h. There are possible negative effects from some possible concurrent wrong behaviors such as smoking or alcohol abuse. There are no investigations about the effect of masturbation in this context. There is a need to clarify the effects of sexual activity on competition performance. The present evidence suggests that sexual activity the day before competition does not exert any negative impact on performance, even though high-quality, randomized controlled studies are urgently needed.

## Background

In ancient times, abstinence was considered the best method to ensure athletic performance and communion between body and spirit. Roman and Greek educators believed that great sacrifices could sustain success. This is probably the main reason why many coaches support the importance of sexual abstinence before sports competition (Oman et al., 2003), believing that sexual frustration leads to increased aggression. They believe that ejaculation draws testosterone away from the body (Krieger, 1997), reducing aggression and muscle strength.

However, the relationship between sexual activity and athletic performance is still controversial. The first scientific publications date back several decades, when some psychological and physiological aspects related to sexual activity were considered in the sport environment (Anshel, 1981). Nevertheless, no controlled data are available about the possible role of masturbation or climax. The role of sexual activity on sport performance has not anyway been examined in a scientific fashion, using a rigorous, reproducible approach, and few studies have been specifically dedicated to this matter up to now (Cooper, 1975).

The relationship between sexual hormones and physical function has been on the contrary studied for both power and endurance performance (Johnson, 1968; Hengevoss et al., 2015). Few authors studied the effects of sexual activity the night before a competition, and most coaches believe that it can have a negative influence on athletic results because of excess energy expenditure (Ferraz and Costa, 2014). The problem it is not yet sufficiently clarified, though the energy expenditure of sexual activity remains low (Krieger, 1997; Baume et al., 2006; Ferraz and Costa, 2014). The lack of sleep associated with such activity may however be remarkable, and contribute to greater energy loss and a reduction in sports performance. It is not yet clear for how long this hypothetical negative impact would last. Some differences on the effects on sports activity could be hypothesized when masturbation or sexual intercourse are considered. The relationship between sexual activity and sports is indeed complex and mutual. Butt (1990) demonstrated that sexual activity could be interpreted as a sort of physical activity, positively impacting on health and wellbeing.

This systematic review evaluates the current scientific evidence in the field of the sexual activity of athletes before sports competition.

## Methods

### Information sources

The current systematic review was conducted following the Preferred Reporting Items for Systematic Reviews and Meta-analysis (PRISMA) guidelines (Liberati et al., 2009). A specific checklist and an *ad-hoc* algorithm with the screening questions were designed and pilot tested within a subset of studies before implementation. The following databases/thesauri were extensively searched from inception: namely, PubMed/MEDLINE, ISI/Web of Science, the Cochrane Collaboration Database, Cochrane Library, Evidence Database (PEDro), Evidence Based Medicine (EBM) Search review, National Guidelines, ProQuest, and Scopus. We used a string made up of the following key words with proper Boolean connectors: coitus, sexual intercourse, sexual activity, sexual climax, orgasm, masturbation, abstinence, sports, competition, exercise, physical activity, strength, speed, endurance, and performance. Truncated words with wild card option and Medical Subject Headings (MeSH) terms were used when appropriate. The platform “Primo Central Ex Libris UNO per tutti” was used.

### Screening strategy

The studies were independently screened by two reviewers (LS and GG), to avoid any bias. Disagreement was assessed using κ statistics, and resolved through discussion; a third reviewer (NLB) was involved if necessary.

### Eligibility criteria

All manuscripts initially considered relevant by title and abstract were eligible for inclusion. The full text of the manuscripts was obtained to ascertain whether they satisfied the following inclusion criteria, detailed according to the PICO standard:

P (population): athletes (at any level, national and international and practicing any sports discipline);
I (intervention/exposure): having sexual activity before a competition/match;
C (comparison): studies comparing athletes reporting sexual activity vs. athletes not having sexual intercourse before a competition/match, where such comparison has been performed;
O (outcome): impact of sexual activity on performance.

Other inclusion criteria concerned the design (original primary articles of any type—case report, case series, observational study, randomized controlled trial, etc.), language (all languages available), and time filter (none applied).

In addition, to broaden our research, the references section of the selected articles was searched by hand to try and identify other relevant articles. Also, target journals were hand-searched. Further, to identify as many potentially relevant manuscripts as possible, a gray literature search using Google Scholar was performed. Finally, a search of theses and dissertations databases was performed.

The search strategy is detailed in Table 1.

**Table 1:** **Details of search strategy used in the current systematic review**.

**Search strategy items**	**Details**
Used keywords	(coitus OR “sexual intercourse” OR “sexual activity” OR “sexual climax” OR orgasm OR masturbation OR abstinence) AND (sports OR athlete) AND (competition OR exercise OR physical activity OR strength OR speed OR endurance OR performance)
Searched databases	PubMed/MEDLINE, ISI/Web of Science, the Cochrane Collaboration Database, Cochrane Library, Evidence Database (PEDro), Evidence Based Medicine (EBM) Search review, National Guidelines, Scopus, ProQuest Research Library, ProQuest Health and Medical Complete, ProQuest Nursing and Allied Health Source, ProQuest Science Journals, ProQuest Health Management, Google Scholar
Inclusion criteria	Original primary article meeting with the established PICO criteria
Exclusion criteria	Editorial, letter to editor, commentary, opinion, expert opinion, review (of any type), article not pertinent with the review question(s)
Time filter	None applied
Language filter	None applied
Target journals	Asian Journal of Sports Medicine; British Journal of Sports Medicine; Clinical Journal of Sport Medicine; International journal of sports medicine; Journal of Sports Sciences; Medicine and Science in Sports and Exercise; Perspectives on Sexual and Reproductive Health; Professional Psychology: Research and Practice; Research in Sports Medicine; Sex Roles; Sports Medicine; The American journal of sports medicine; The Journal of Sexual Medicine; The Journal of sports medicine and physical fitness; The Physician and Sportsmedicine

### Appraisal of study quality

Two reviewers were contents experts (LS and GG), and one reviewer (NLB) was an experienced biostatistician/epidemiologist. The contents experts only assessed potential publications with respect to the appropriateness of the research questions tested. The biostatistician only evaluated the appropriateness of the methods employed. Disagreement was resolved by consensus.

### Strength assessment of the scientific evidence

The strength of the body of evidence was assessed using the Grading of Recommendations, Assessment, Development and Evaluations (GRADE) evidence system (accessible at http://clinicalevidence.bmj.com/x/set/static/ebm/learn/665072.html).

## Results

The initial search produced 512 references: 95 from PubMed/MEDLINE, 120 from Scopus, 98 from ISI/Web of Science, 52 from ProQuest Research Library, 51 from ProQuest Health and Medical Complete, 29 from ProQuest Nursing and Allied Health Source, 16 from ProQuest Science Journals, 14 from ProQuest Health Management, 7 from the Cochrane Library, 11 from PEDro, 17 from the National Guidelines, 2 from the EBM. After removing duplicates, the search yielded 142 unique results. One further study was added after mining Google Scholar.

**Figure 1:**
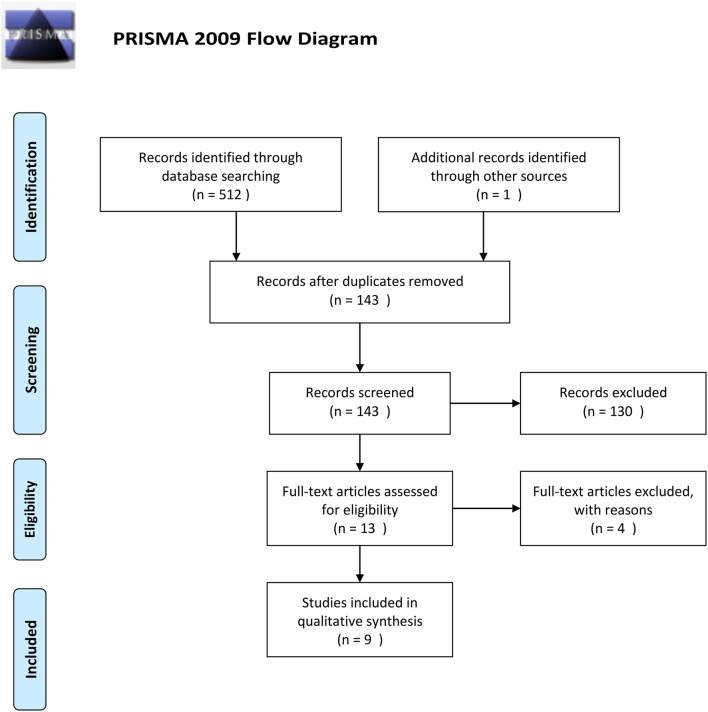
**Flow diagram of the current systematic review conducted according to the Preferred Reporting Items for Systematic Reviews and Meta-analysis (PRISMA) guidelines**.

One hundred and thirty studies were rejected because of off-topic abstracts, failure to fulfill the inclusion criteria, or both. After reading the full text, four studies were excluded, not being relevant to the subject at hand. Finally, nine studies were included in the current systematic review. Fort further detsails, the reader is referred to Figure 1 and Table 2.

**Table 2:** **Details of the studies included**.

**Authors**	**Study group**	**Age**	**Study design**	**Tests used**	**Aerobic power**	**Strength power**	**Psychological hormonal findings**
Boone and Gilmore, 1995	11 sedentary male	Not specified	Cross-over	Treadmill test	O_2max_, DP	Not tested	No differences
Dabbs and Mohammed, 1992	11 M/F	Not specified	Not controlled	Blood sample	Not tested	Not tested	No modifications in blood testosterone levels
Fisher, 1997	166 varsity athletes (*n* = 83 football; *n* = 73 baseball)	Not specified	Controlled	General investigation	Not tested	Not tested	Religion has an impact on sexual behavior, depending also on the kind of sport (team vs. individual)
Frauman, 1982	144 subjects + 78 undergraduate subjects	Not specified	Exploratory	Questionnaire	Not tested	Not tested	Physical exercise modifies sex behavior in a statistically significant way (Pearson's correlation *p* < 0.001)
Johnson, 1968	14married Female Athletes	24–49 years, mean 28 years	Randomized	Hand grip–dynamometer	Not tested	No modifications	Not tested
Meston and Gorzalka, 1995	Female	Not specified	Not controlled	Vaginal photoplethysmograph	Not tested	Not tested	Acute exercise increase arousal in female
Pour et al., 2013	Not tested (brief revision of literature	Since 18 years	Revision	Anonymous questionnaire, Mental Health	Mental Health	Not tested	Positive psychological impact
Sztajzel et al., 2000	15 male athletes high level	20–40 years	Randomized	Cycloergometer Ex Test	HR	Not tested	No significant differences
				Mental Test			
Vouyoukas, 2011	8 participants	Not specified	Thesis, observational	Cardiovascular and muscular test	VO_2max_, DP	Hand grip flexibility,	No differences

*AP, Aerobic Power; SP, Strength Power; H, Hormonal; Psy, Psycological; DP, Double Product; HR, Heart Rate*.

### Study selection

From the general point of view, none of the studies selected have approached this aspect systematically, and there is no evidence of a methodical investigation of the possible differences by gender, or intensity, or the type of the sports practiced. In most of the few manuscripts identified, males are more frequently investigated than females. Some specific aspects emerge however in a study from the analysis of the female athletes population with respect of male. The authors (Johnson, 1968) studied a group of 14 female former athletes, ages 24–49 years, to test the impact of sexual intercourse on strength performance, and some features of the muscular and aerobic performance were found. The study was conducted in two different sessions: the first time the morning after coitus the previous night, and then at least 6 days after the last coitus (Johnson, 1968). Muscle strength was tested using dynamometry: no influence of sexual intercourse on muscle strength was found (Johnson, 1968). Similar data regarding the effects of coitus on muscle strength were confirmed in 1995 in another study despite involving a population of male sedentary subjects (Boone and Gilmore, 1995). In this study, aerobic performance was studied, and the possible impact of sexual intercourse was evaluated using cyclo-ergometry: sexual intercourse does not impact negatively when it takes place at least 10 h before the competition. A negative effect, however, occurs if there is an interval of less than 2 h between sexual intercourse and the test (Boone and Gilmore, 1995). The effects of sexual intercourse were studied in terms of possible modifications of the cardiovascular variables such as maximal aerobic power and oxygen pulse (Boone and Gilmore, 1995). All the variables considered were not influenced by sexual intercourse, which did not decrease maximal exercise performance. Significant differences were achieved for post-effort heart rate (HR) values at 5 (*p* < 0.01) and 10 min (*p* < 0.01) during the recovery phase of a morning test 2 h after sexual activity. These differences disappeared during the recovery phase of the afternoon stress test performed approximately 10 h after sexual intercourse (Boone and Gilmore, 1995). Despite the major differences found in the recovery phase, where higher values of HR were found 2 h after sexual intercourse, no significant differences were found in workload achieved and in mental concentration of the athletes. The data support the role of sexual activity in activating the sympathetic system, and the same mechanism can be advocated in increasing testosterone blood levels after sexual activity.

The possible impact of sexual intercourse was evaluated in well-trained male amateur runners. Sztajzel et al. (2000) evaluated, in addition to the impact of sexual intercourse, also the possible impact of additional four incorrect health behaviors on marathon performance. The study supports the potential beneficial effect of sexual activity on running performance, and it underlines the negative impact of other incorrect lifestyle habits such as smoking and alcohol intake (Sztajzel et al., 2000). However, the number of subjects investigated was small, and some other variables such as sleeping, nutrition habits and recreational activities need to be considered to be able to speculate accurately.

Sexual intercourse may have a negative impact on athletes' strength and muscular performance (McGlone and Shrier, 2000), estimated by the handgrip test. Strength was tested the morning after nocturnal coitus, and the results were compared undertaking the same test after at least 6 days of sexual abstinence (McGlone and Shrier, 2000). Handgrip strength was not affected by sexual activity the night before each test. McGlone and Shrier (2000) reported the possible influence of sexual activity on coordination and maximal aerobic power. Other unreferenced and referenced studies investigated the influence of sexual intercourse on athletes' performance in a group of 14 married males, with no evidence of a negative effect on VO_2_ max and coordination (Nemec et al., 1975; Thornton, 1990). The authors suggests investigating in parallel some other variables, particularly in the presence of prolonged abstinence, to exclude a possible negative impact (Thornton, 1990). Despite no evident impact of sexual intercourse on athletes' performance, the sample investigated was too small to allow generalization.

Few studies specifically distinguish possible difference in the impact of sexual activity in different types of sports. Fisher (1997) analyzed the effects of different sexual behaviors in two different sports, soccer and baseball. Soccer players practiced abstinence more frequently than baseball players, and the possible positive results on their performance may have resulted from more frequent abstinence before competition (Fisher, 1997). In addition, considering the potential effect of regular sport activity on blood hormones regulation, several modifications should be expected in hormone blood levels. Before competition, a rapid hormonal variation in individuals can be linked to variation in physiology, and can also be species related (Wobber et al., 2010). Indeed, experiments in chimpanzees, as a model close to humans, demonstrate rapid changes of testosterone and cortisol during competition (Wobber et al., 2010). These experiments also highlight inter-individual and inter-species differences: one individual's own perception of a given situation may induce different hormonal responses.

The issue of testosterone level after sex is debated: some studies found no changes in testosterone levels after sex (Hengevoss et al., 2015) in males. The study reports some examples of investigations where testosterone blood levels were tested at different times following sexual intercourse. Sexual activity does not affect testosterone levels, in the short or long term. In that study, some athletes showed worse or improved performance after intercourse, but no systematic effect of sexual intercourse was demonstrated.

Salivary testosterone concentrations were measured in male and female members of four heterosexual couples on 11 evenings before and after sexual intercourse, and on 11 evenings when no intercourse took place. Testosterone levels increased across the evenings when the couples engaged in intercourse, and decreased when they did not. The data suggest that sexual activity affects testosterone more than initial testosterone affects sexual activity (Dabbs and Mohammed, 1992).

On the contrary, a complete different point of view is evident from international competitions such as the Olympics: the traditional idea of the importance of sexual abstinence before competition had generally been supported (James, 1990; Anderson et al., 2001). More recently, the opinion of most athletes and coaches is shifting toward the fact that sex itself has little or no impact on sporting performance, on the basis of the possible psychological motivations related to normal sexual behavior (Chidley, 1996; Pupiš et al., 2010). The authors underline generally that sexual activity does involve physical activity (Gordon, 1988), and psychological and emotional involvements are part of sexual behavior. Sexual activity has a relaxing effect, and the frustration to limit one's own sexual desire is probably more detrimental than to actually engage in sexual activity.

McGlone and Shrier (2000) demonstrated no evidence of a direct negative impact of sexual intercourse in sports performance: the authors consider some specific psychological aspects (McGlone and Shrier, 2000). For example, considering the inverted “U sport psychology hypothesis,” the frustration from prolonged abstinence can promote the desire to better athletic activity, while being sufficiently fulfilled sexually can reduce such desire for better sports performance (Thornton, 1990). There is an optimal level of alertness/anxiety before a competition, and therefore sex may produce a relaxing distraction effect the night before competition.

A special aspect of sex behavior regards therefore the psychological impact of sexual activity on sport performance (Frauman, 1982). The authors confirm the scarcity of data available, the diversity of opinions, and the necessity to carefully study the relationship between sexual behavior and sport. Sexual intercourse expends generally only an average of 25 calories, corresponding to walking up two flights of stairs. Even from the psychological point of view, the sports world leans toward abstinence, believing that sexual activity can impair sport performance (Vouyoukas, 2011). Nevertheless, most studies in this field conclude that sexual expression may have a strong relationship with quality of life (Sayfollahpour et al., 2013). Sexual satisfaction is directly associated to a higher level of quality of life, while prolonged abstinence can be associated to depression (Vouyoukas, 2011). There is a strong relationship between regular sex and mental health levels, comparing the tendency to maintain abstinence to a sort of homophobia. This aspect is far from the evaluation of the specific aspect where sexual intercourse is directly linked with an increase or decrease of performance in athletes. Some other possible influences on sports performance of different sexual habits, such the effects of viewing pornographic films, are reported (Pour et al., 2013). The findings suggest activation of the sympathetic system associated to physiological arousal (Meston and Gorzalka, 1995). The authors support the strong relationship between sexual activity and physical exercise. Some studies investigated whether exercise can be used as therapy in case of reduced arousal, and have shown a positive association between regular sexual activity and increased blood testosterone levels (Kraemer et al., 1976).

Sexual abstinence has been indirectly investigated in terms of the potential impact on well-being. A special investigation has reported the effects of sexual activity on maintaining well-being, with no evidence of a negative impact of sexual activity on sport performance (Levin, 2007). In addition, more extensively, a doctoral thesis evaluated the influence of sexual activity on athletic performance, comparing sexually active subjects with subjects who practiced abstinence. There were no substantial differences in terms of physiological variables: (heart rate and blood pressure), sport specific parameters (all out test, upper and lower limb strength, reaction time, hamstring flexibility), and biochemical variables (testosterone, cortisol, and glucose levels; Vouyoukas, 2011).

There are marked differences between individual and team sports athletes: team sports athletes were more prone to pre-marital sexual intercourse, and thus had more sexual partners, while individual sports athletes were more conservative in this respect (Pour et al., 2013).

In general, there is a global positive impact of sex the night before competition on athlete's performance. Especially from the psychological point of view, sex has a relaxing effect, which may help to relieve competition stress in endurance (marathon) or concentration (archery or pistol shooting) sports. Some coaches support the role of relaxation in improving coordination and peak athletic performance.

## Discussion

The impact of sexual activity in sport, especially before sport competition, has been studied for the last 60 years. Many questions relate to athletes' performance, and several are not yet solved. Considering that top sports competition is undertaken by young adults, and therefore the expectancy of success is high, it is not surprising that the prospect that decreased athletic performance from sexual activity has been the object of interest by governing bodies, officials and coaches (Catania and White, 1982). Few authors have studied this aspect of sports participation in a systematic fashion. Many studies did not use appropriate scientific methods on the possible impact of sexual intercourse; it appears that there is no evidence of a negative impact in males and females. It appears important to maintain an athlete's own sexual activity in terms of normal physiological behavior, and to avoid possible association with incorrect lifestyle habits, such as tobacco, alcohol or drugs abuse. At least a few hours should pass between sexual intercourse and sports competition. The available investigations have not considered the possible role of confounding factors. There are no trials conducted with acute exercise and laboratory test to study the possible hormonal modifications induced by sexual intercourse in this context. Also, the design of the available studies is not strong, and probably they do not reflect real life situations. In some sports and in several cultures, coaches continue to support sexual abstinence before sports competition. On the other hand, in major competitions such as the Olympics and the Commonwealth Games, contraceptive devices are freely available, acknowledging that sexual activity is normally practiced by the athletes during the games.

### Areas of agreement

The idea of a possible benefit of abstinence before sports competition is ancient, based on anecdotal evidence, and not sufficiently supported by the recent scientific literature. Only one study (Fisher, 1997) promotes abstinence on the basis of a possible influence of frustration in maintaining high level sports performance. On the contrary, most studies identified support the absence of negative effects of sexual activity on sports performance (James, 1990; Thornton, 1990; Pupiš et al., 2010). Others studies often underline the importance to maintain sexual experiences within an athlete's normal sex life (Kraemer et al., 1976; Frauman, 1982; Hengevoss et al., 2015). Any possible association of sexual activity with incorrect lifestyle habits, such as abuse of alcohol or smoking, can produce adverse effects and worse performance.

### Areas of controversy

From the present review, the major area of controversy concerns the principal aspect of the sexual intercourse and therefore if it can be considered correct on not, in terms of sports performance, to permit to have sexual intercourse before the sport competition. While some coaches continue to highlight the importance of abstinence, the literature does not support this belief. From the present review, only low powered studies of relatively poor scientific design have investigated the impact of sexual activity using specific tests to evaluate the effects on aerobic power and strength (Boone and Gilmore, 1995). No data are available about the possible diverse effects of sexual activity on different sports. Some data are available on the possible psychological impact of sexual activity on athletic performance (Catania and White, 1982; Vouyoukas, 2011) and all support a positive effect or at worse no impact.

### Growing points and areas timely for developing research

At present, there has been little methodologically well-performed research on this topic. Also, given the variety of sports, and their different metabolic and situational differences, generalization is not possible. Most studies seem to have focused on male athletes: at present, there is a growing population of female athletes, and it is unclear whether gender differences could be evident. Finally, the attitude to sexual activity changes according to different cultures, countries and religions: this is a further variable that should be investigated.

## Conclusions

From the present review, doubts remain regarding the possible negative impact of sexual activity the night before competition. This aspect is considered important in sports, but there is insufficient evidence of the possible specific detrimental effect on the sports performance. In addition, no exhaustive data are available about the possible impact of the sexual activity on different kinds of sports, in terms of endurance or resistance performance, or in terms of team or individual sports. There are few scientifically sound data about the effect masturbation (Catania and White, 1982) on sports performance. This is a specific aspect not yet investigated in a scientific fashion. Some anecdotal reports support positive experiences in competitive athletes. Cultural and religious beliefs influence the approach to sex and sport. The sexual sphere is individual, and in this context athletes should likely feel free to live their sexual activity in complete freedom. The present review demonstrates that sex activity in sport is poorly investigated in both males and females. However, the data available do not really support the misconception that sex activity can produce a negative effect on the athlete's performance. Anecdotal experiences sustain, on the contrary, a positive effect of performance if sexual activity is undertaken at least 10 h before sports competition, and particularly if it is not associated to incorrect life style habits such alcohol and drugs abuse, and smoking. Future investigations should clarify in greater depth some specific aspects related to ethnical, gender and sport differences.

## Author contributions

LS conceived, designed and wrote the paper; GG revised the final version of the paper; JP and NB revised and contributed to improve the statistical analysis and approach of the systematic review. NM continuously supervised the manuscript. All authors read and approved the final version of the manuscript.

### Conflict of interest statement

The authors declare that the research was conducted in the absence of any commercial or financial relationships that could be construed as a potential conflict of interest.
